# Time trend by region of suicides and suicidal thoughts among Greenland Inuit

**DOI:** 10.3402/ijch.v74.26053

**Published:** 2015-02-19

**Authors:** Peter Bjerregaard, Christina Viskum Lytken Larsen

**Affiliations:** 1 Centre for Health Research in Greenland, National Institute of Public Health, University of Southern Denmark, Øster Farimagsgade, Copenhagen, Denmark; 2 Greenland Centre for Health Research, University of Greenland, Nuuk, Greenland

**Keywords:** suicides, suicidal thoughts, Greenland, regional variation, temporal trend

## Abstract

**Background:**

Suicides remain a major public health problem in Greenland. Their increase coincides with the modernization since 1950. Serious suicidal thoughts are reported by a significant proportion of participants in countrywide surveys.

**Objective:**

To analyze the time trend by region of suicides and suicidal thoughts among the Inuit in Greenland.

**Design:**

Data included the Greenland registry of causes of death for 1970–2011 and 2 cross-sectional health surveys carried out in 1993–1994 and 2005–2010 with 1,580 and 3,102 Inuit participants, respectively.

**Results:**

Suicide rates were higher among men than women while the prevalence of suicidal thoughts was higher among women. Suicide rates for men and women together increased from 1960 to 1980 and have remained around 100 per 100,000 person-years since then. The regional pattern of time trend for suicide rates varied with an early peak in the capital, a continued increase to very high rates in remote East and North Greenland and a slow increase in villages relative to towns on the West Coast. Suicidal thoughts followed the regional pattern for completed suicides. Especially for women there was a noticeable increasing trend in the villages. The relative risk for suicide was highest among those who reported suicidal thoughts, but most suicides happened outside this high-risk group.

**Conclusion:**

Suicide rates and the prevalence of suicidal thoughts remain high in Greenland but different regional trends point towards an increased marginalization between towns on the central West Coast, villages and East and North Greenland. Different temporal patterns call for different regional strategies of prevention.

Suicide has been recognized as a major public health problem in the post–World War II generations in Greenland as in other circumpolar indigenous populations. It is an indicator of poor mental health especially among young men. In 2006, an epidemiological overview of data until 1999 concluded that although the increase in suicides coincided with the modernization after 1950, the time trend was different in different regions of the country and that it was not possible to pin point any specific components of modernization as the main causes of the increase in suicide rates (1).

More recent studies include a psychological autopsy study of 7 cases of suicide in East Greenland which showed that even though the act itself was impulsive, the suicides were not inexplicable and all happened in known risk groups (2). Although most authors have concentrated on social risk factors, latitude and season may play a role since suicides were more common in the summer months, especially at high latitudes (3, 4). The incidence of suicide attempts by overdose of medications was high in Nuuk compared with England and Wales, especially among young women (5). Alcohol intoxication was present in 50% of emergency visits due to suicide attempts in the Greenland health care system (6). In a circumpolar study, the prevalence of suicidal thoughts among young women was higher in Greenland and Alaska than in Norway and Sweden (7).

In the United States, suicide rates among indigenous people were highest in Alaska (8) and suicidal behaviour was most common among single, unemployed Alaska Natives who had not completed high school and who had a history of substance abuse (9). In Canada, the Inuit youth were seen as victims of the colonial era that destabilized the kin-based social organization of the Inuit (10, 11). A large psychological autopsy study of suicide among Inuit in Nunavut is underway (12).

There is no sign of an abatement of the suicide epidemic in circumpolar indigenous populations despite multiple attempts at prevention. Recently, the circumpolar public health community has shown a renewed interest in concerted prevention of suicides epitomized by the international conference on Hope and Resilience in Nuuk in 2009 (13) and a recent joint CIHR/Arctic Council initiative regarding “The Evidence-Base for Promoting Mental Wellness and Resilience to Address Suicide in Circumpolar Communities” (14).

The purpose of the present article is to present an updated analysis of the time trend by region of suicides and suicidal thoughts among the Inuit in Greenland, a population for which exceptionally good data are available both cross-sectionally and in a time perspective of more than 40 years.

## Greenland

The total population of Greenland is 57,000 of whom 90% are ethnic Greenlanders (Inuit). Genetically, Greenlanders are Inuit (Eskimos) with a mixture of European, mainly Scandinavian genes (15). They are genetically and culturally closely related to the Inuit/Iñupiat in Canada and Alaska and, somewhat more distantly, to the Yupiit of Alaska and Siberia. Under Danish colonial system, from 1721 to 1953, Greenland has had home rule since 1979 and a self-governing status since 2009. Urbanization started in the early 20th century and has increased rapidly since the 1950s. In 1951, 68% of the population lived in villages with less than 500 inhabitants; by 2010, this proportion decreased to 15%.

Greenland's 80 communities are all located on the coast. The communities are divided into towns and villages. A town is defined historically as the largest community in each of the 17 districts. In 2010, the population of the towns varied between 469 and 5,460 with the capital, Nuuk, having 15,469 registered inhabitants and probably a fair number of unregistered ones, while that of villages varied from less than 10 to around 550. Located in the towns are the district school(s), health centre or hospital, church, district administration and the main shops. These institutions are absent or present to a much smaller extent in villages. The capital, Nuuk, has a population 3 times that of the second largest town and despite its small size has many of the characteristics of a northern capital such as central government offices, a university and other post-secondary teaching institutions, the central hospital for Greenland, and so on. The most remote communities in Greenland are situated on the East Coast and in the far north. They were colonized much later than the central West Coast and suffer from being remote, having dialects that differ considerably from Central West Greenlandic, lower income and less employment opportunities.

## Methods

Information on suicides was obtained from the Greenland registry of causes of death which covers the period since 1968 and is regularly updated. Information from death certificates is validated against the Central Population Registry. During the period under consideration for the present analyses (1970–2011), a total of 1,678 persons born in Greenland (a proxy for Inuit ethnicity) were registered with a diagnosis of suicide (ICD8 and ICD10) among a total of 16,648 registered deaths. During the past 7 years, the number of suicides independently recorded by the police was similar to those in the registry of causes of death (330 and 328, respectively) (16).

Survey data were collected by interview and self-administered questionnaires as part of 2 general population health surveys. In 1993–1994, data (N=1,728/1,580 Inuit) were collected in 38 towns and villages in Greenland as part of a general population health survey with a participation rate of 57% (17). In 2005–2010, data (N=3,253/3,102 Inuit) were collected in 21 towns and villages as part of a general population health survey with a participation rate of 67% (18). Questionnaires were developed in Danish language, translated into Greenlandic, back translated and revised. The questions were originally adapted from a Danish health interview survey for the 1993–1994 study (17, 19) and subsequently further developed with the inclusion of questions from surveys among the Inuit in Canada and Alaska. Interviews gave information about socio-demographic factors, self-rated health and disease, and lifestyle, including diet, physical activity and smoking. Information about alcohol use and suicidal behaviour, among other topics, was obtained from self-administered questionnaires. Interviews were conducted in the language of choice of the participant, most often in Greenlandic, by native Greenlandic-speaking interviewers who had been trained in the study procedures. The questionnaires were available in Greenlandic and Danish.

Information on suicidal thoughts was obtained from the self-administered questionnaire as part of these surveys. The question which has been used since 1993 runs: “Have you ever seriously considered suicide? If yes, was this within the last year?” The variable used for the present analyses was suicidal thoughts within the past year. Of 4,212 participants who filled out the self-administered questionnaire in 1993–1994 and 2005–2010, 3,808 (90%) answered this question.

Register data for suicides was obtained from Bertelsen (20) for 1901–1933, annual reports from the Chief Medical Officer in Greenland (21) for 1950–1969 and our in-house register of causes of death in Greenland for 1970–2011. Figure 5 illustrates a fictive cohort based on information from the 1993–1994 survey, the register of causes of death and life tables from Statistics Greenland (22).

### Statistics

Analyses were performed with standard software: IBM SPSS v. 21 (22) and Excel 2010. Information on population size was obtained from Statistics Greenland (23) and from the 1970 census (24). Standardization for age and sex to World Standard Population 2000–2025 (25) and calculation of rates was carried out in Excel. Statistical tests included Chi-square test (Tables I and II) and Univariate General Linear Models (UNIANOVA procedure of SPSS) (Fig. 4). In Fig. 5, suicide rates were calculated from a follow-up in the registry of causes of death of the participants in the 1993–1994 Population Health Survey and subsequently the number of suicides in the cohort was estimated by these rates. Confidence intervals were derived from tables of the Poisson distribution.

**Table I:** Suicides among Inuit in Greenland 1901–2011

	N	N per year	Crude rate per 100,000 person-years
1901–1930	10	0.3	2.4
1924–1933	5.3	0.5	3.5
1951–1954	1	0.1	0.5
1955–1959	0	0.0	0.0
1960–1964	23	4.6	14.4
1965–1969	33	6.6	18.1
1970–1974	56	11.2	28.3
1975–1979	91	18.2	45.0
1980–1984	203	40.6	96.5
1985–1989	261	52.2	117.2
1990–1994	238	47.6	101.0
1995–1999	245	49.0	100.3
2000–2004	227	45.4	91.2
2004–2009	211	42.2	84.0
2010–2011	111	55.5	110.4
1970–2011	1,678	40.0	87.7
Capital	303	7.2	86.6
Towns in West Greenland	837	19.9	81.2
Villages in West Greenland	222	5.3	61.4
East and North Greenland	307	7.3	187.5
			p<0.0001*

Source: Bertelsen 1935; reports of the Chief Medical Officer for Greenland; Greenland registry of causes of death.

* p for difference between regions (Chi-square).

**Table II:** Suicides and suicidal thoughts by age and sex among Inuit in Greenland 2000–2011

	Suicidal thoughts last year per 1,000 participants		Suicides per 100,000 person-years	
		
	2005–2010		2000–2011	
		
Age	Men	Women	Men	Women
10–14			45	32
15–19	91	188	297	152
20–24	136	183	416	97
25–29	128	144	251	63
30–34	107	108	147	84
35–44	71	100	139	44
45–54	38	70	105	51
55+	19	14	74	24
	P<0.0001	P<0.0001	P<0.0001	P<0.0001

p-Values for differences among age groups (Chi-square).

### Ethical considerations

The studies were ethically approved by the Commission for Scientific Research in Greenland. Participants gave their oral (1993–1994) or written consent after being informed about the study orally and in writing.

## Results

Information about suicides in the Inuit population of Greenland is available from a number of sources from the beginning of the 20th century (Table I). The recorded crude incidence increased from 1960 to 1980 and has remained relatively constant since then. For the period 1970 to 2011, the information allows a detailed analysis according to age, sex and region. Seen over this 40-year span, the crude suicide rates have been a little lower than average in the villages in West Greenland and considerably higher in East and North Greenland.

**Fig. 1:**
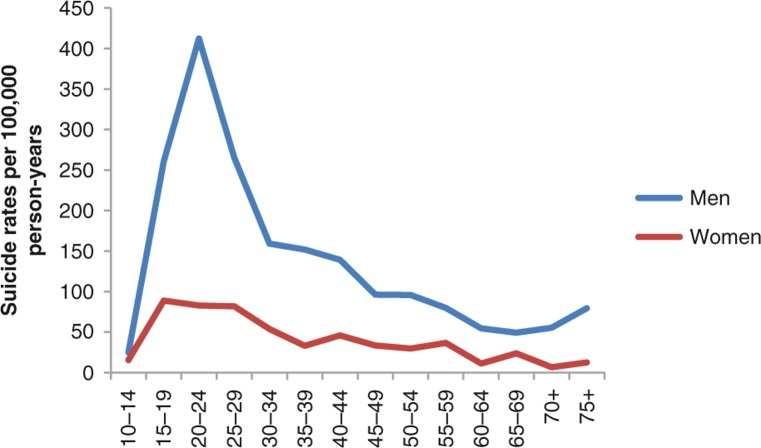
Suicides per 100,000 person-years by age group among Inuit men and women in Greenland 1970–2011. N=1,687.

Suicide rates for males were considerably higher than rates for females and showed a distinct peak in the 20–24 age group with the rates trailing off later in life (Table II and Fig. 1). In females there was also a peak in the young age groups but the pattern was not as conspicuous as in males.

**Fig. 2:**
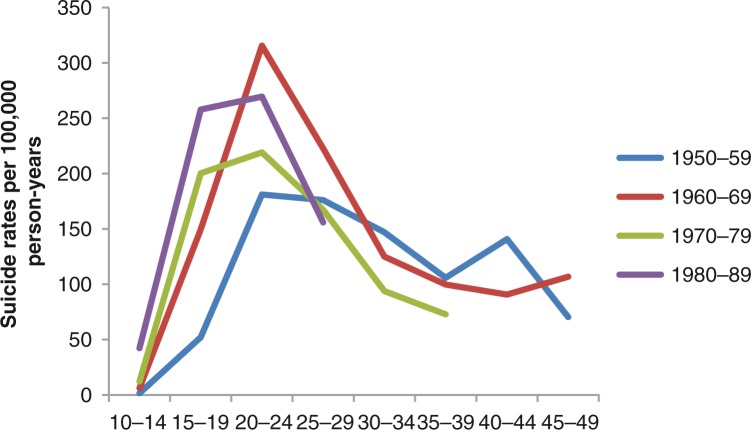
Suicides per 100,000 person-years by age group in four 10-year birth cohorts. Inuit in Greenland born from 1950 to 1989. N=1,346.

Although this age pattern was discernible in 4 birth cohorts, a noticeable change took place over time (Fig. 2). A visual inspection showed a leftwards displacement of the curves with increasing birth cohort which indicates that those who committed suicide became younger in each succeeding birth cohort from 1950–1959 to 1980–1989. The peak at age 20–24 was lowest in the 1950–1959 cohort and highest in the 1960–1969 cohort. For the 30+ age groups, the suicide rates were similar in all birth cohorts.

**Fig. 3:**
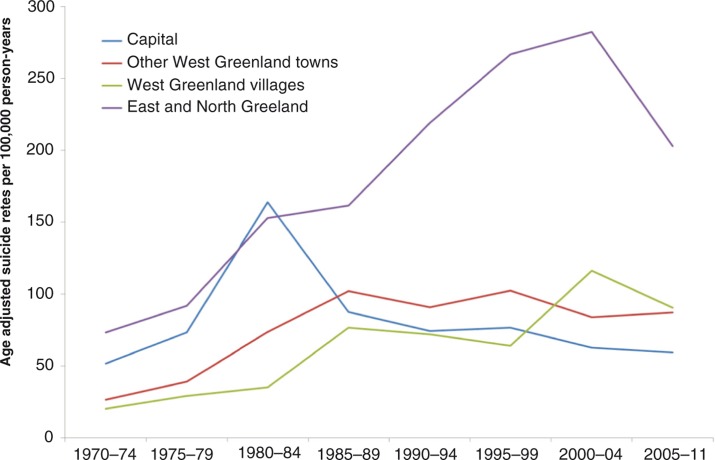
Temporal trend of suicides in 4 regions of Greenland. Inuit in Greenland. Rates per 100,000 person-years standardized to World Standard Population 2000–2025. N=1,669.

A visual inspection of Fig. 3 shows that the temporal trend of suicides differed considerably by region. In the towns of West Greenland, the temporal pattern was similar to that of the whole country, that is, an increase until the late 1980s followed by stagnation of rates. In contrast, the capital had an early rise in rates in 1980–1984 followed by a decrease and rates have been lower than in the other towns in West Greenland since 1985–1989. From the start, the suicide rates in the villages in West Greenland were relatively low but a steady increase has brought them at the same level as those of the towns. Finally, the suicide rates in East and North Greenland have remained the highest of all since 1985, recently more than twice as high as rates in West Greenland although there was a decline in the most recent period. Since 1985, the rates have differed little among the 3 regions in West Greenland but since 2000 the rate for the capital was below those of the rest of West Greenland (p=0.004) while the rate in East and North Greenland was higher (p≤0.0001). The pattern was similar for men and women.

Suicides and suicidal thoughts (within the past year) had similar age patterns with peaks for men at age 20–29 and for women at age 15–24 (Table II). While suicide rates were much higher for men at all ages, the prevalence of suicidal thoughts was somewhat higher for women (at most ages). Among participants in the age group 18–29, 28% had ever had serious suicidal thoughts while 25% had attempted suicide.

**Fig. 4:**
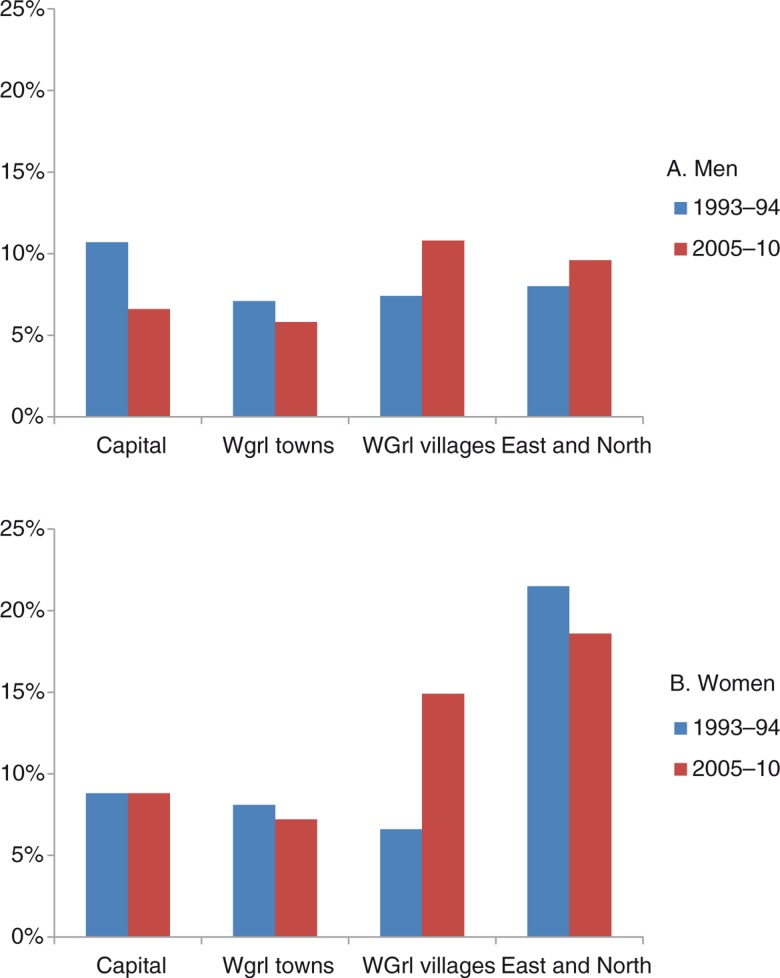
Prevalence of recent (within 1 year) suicidal thoughts in 1993–1994 and 2005–2010 in 4 regions of Greenland. A: Inuit men (N=604 and 1,034, respectively); B: Inuit women (N=664 and 1,214, respectively).

Similar to what was observed for suicide rates, the temporal trend for suicidal thoughts also differed by region but the picture was different for men and women (Fig. 4a and b). The pattern was particularly noteworthy for women (Fig. 4b). In the capital and other towns in West Greenland, the prevalence of suicidal thoughts among women was relatively low (9%) and did not change from 1993–1994 to 2005–2010. In East and North Greenland, the prevalence was high, about 20%, and there was also little change between the 2 surveys. In the villages in West Greenland, however, the prevalence more than doubled from 7 to 15% (p=0.017). The overall regional differences for women were statistically significant in 1993–1994 (p=0.04) and 2005–2011 (p<0.0001). For men, the overall regional differences were not statistically significant in either study.

**Fig. 5:**
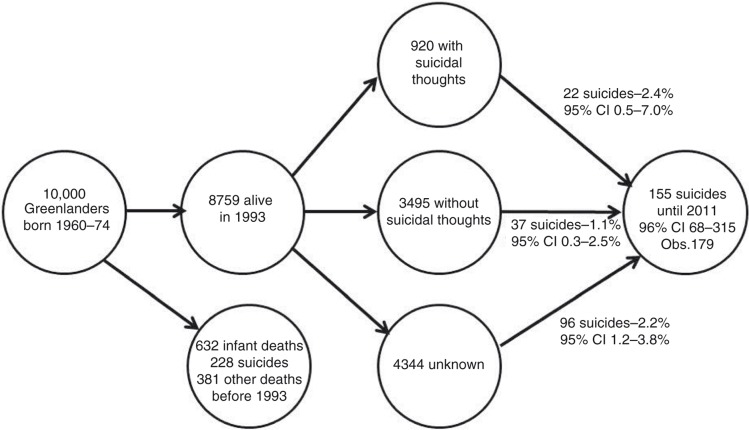
Suicide experience in a fictive cohort of Inuit born 1960–1974.

Figure 5 illustrates the experience with suicidal thoughts and suicides in a cohort of Inuit born in 1960–1974. Most suicides had already taken place before the cohort was invited to participate in a population health survey in 1993. Those who reported suicidal thoughts had the highest risk of later committing suicides (2.4%; 95% CI 0.5–7.0%) but this group contributed with relatively few of the total number of suicides. Those who for various reasons (e.g. declined, not contactable) did not participate in the survey had an almost equally high risk of later suicides (2.2%; 95% CI 1.2–3.8%) and contributed with most of the suicides. Those who reported not having had suicidal thoughts had the lowest risk (1.1%; 95% CI 0.3–2.5%).

## Discussion

The main results show at a countrywide level that after an increase during 1960–1980, the suicide rate remained constant but with different regional patterns. The increase coincided with the coming of age of the generations that grew up after 1950. During this period, Greenland was subjected to a rapid development of infrastructure and a major influx of skilled workers and other cadres from Denmark which in a few years profoundly changed the living conditions in the country. It is reasonable to interpret the social development and the suicide epidemic as being causally related but knowledge about the mechanisms is not readily available. A countrywide concerted strategy for the prevention of suicides was initiated in 2005 (26) but this did not result in lower suicide rates, which were similar in 2000–2004 and 2005–2011 (94.8 and 91.5 per 100,000 person-years; p=0.68). Suicides are, however, the results of a multitude of factors and the absence of a measurable effect on suicide rates is not an indication of an ineffective program because improvements in mental health and living conditions may in a very long perspective lead to reductions in suicide rates. The results of the present study indicate that different regional patterns of development may call for different preventive strategies.

The increase in suicide rates until the 1980s may be explained by the community development as outlined in the introduction but the constant level during the following years is noteworthy given the fact that the community has continued its development; urbanization is increasing, average alcohol consumption is decreasing (23) and the opportunities for communication and travel have increased. One explanation could be that the changes have not included the most central risk factors for suicides such as, for instance, alcohol abuse and sexual abuse in childhood. Another explanation could be that while the prevalence of risk factors decreases the perception of suicide as a solution to a variety of problems is becoming increasingly acceptable.

All deaths in Greenland are recorded by resident physicians in each district and in the case of unexpected deaths or deaths that could be due to intentional injuries, the manner of death is decided on between the physician and the police. The recording of suicides has been regarded as both complete and valid (27). The consistency between the suicide statistics of the police and the Chief Medical Officer is very good but the possibility exists that some suicides hide among deaths without a diagnosis (829 cases; 5% of all deaths) or injuries for which the manner of death is uncertain (147 cases; 0.9% of all deaths). The distinct age and gender distribution of suicides is, however, not present in those 2 groups.

Suicide rates were higher for men than for women. Those who committed suicide became younger over time but in all of four birth cohorts from 1950 to 1989 the suicide rates showed a peak among the 20–24 year old. The prevalence of recent serious suicidal thoughts is higher among women than men but show the same age pattern as completed suicides. The difference in suicide rates between men and women was particularly pronounced in the 20–24 age group. The difference between suicide rates for men and women could be due in part to the choice of suicidal methods since men choose more drastic methods (1, 5). Unfortunately, there is very little information about suicide attempts in Greenland, but a study among Inuit youth in Nunavik, Canada, showed a similar gender pattern with much higher prevalence of suicide attempts among women than among men (28).

Focusing on completed suicides suggests that young men face greater mental health problems than young women, but looking at suicidal thoughts turns the conclusion around and indicates that women more often have mental health problems. The gender differences call for separate analyses for men and women which unfortunately reduces the already low statistical power due to the small populations and the small absolute number of suicides especially among women. As long as patterns are similar for men and women it is acceptable to present results for men and women together.

Although not directly supported by the data of the present study, the early rise and subsequent decrease in suicide rates in the capital may be explained by a combination of early onset of the modernization process followed by generally better living conditions and wealth in the capital. The later increase but sustained very high suicide rates in the remote parts of the country (East and North Greenland) may similarly be explained by a later onset of modernization and continued poor social and economic living conditions.

The difference between West Greenland and East and North Greenland is also found for suicidal thoughts in particular for women. For both genders, but in particular for women, there is a temporal shift in the regional pattern within West Greenland towards a higher prevalence of suicidal thoughts in the villages. Despite a widespread understanding that urbanization is an important reason why the Greenlanders have become alienated, Trondheim (29) concluded that urbanization is part of the contemporary lifestyle in Greenland and is no longer experienced as an alienating factor by young people. It is in this context noteworthy that the negative depiction of urban life and urbanization was already present in the 1800s (29). The trends in suicides and suicidal thoughts shown in this paper may indicate that urban life is becoming preferable to village life among young people. This is easy to understand; there isn't much to do in the villages and in towns, especially in Nuuk, and you have a greater freedom to choose (29). The villages in West Greenland are, however, not a homogenous group of communities and part of the explanation may be that the villages included in the 2 surveys in 1993–1994 and 2005–2010 were not identical.

Reporting serious suicidal thoughts in a survey is significantly associated with a later suicide but this high-risk group contributed with only 14% of suicides while those who did not participate in a population health survey although they were part of the sample accounted for two-thirds. Suicidal thoughts is accordingly not a good screening tool but should be regarded as a general measure of poor mental health besides being a risk factor for suicide.

The strengths of the study include the comprehensive information on all suicides in the country over a 40-year period in combination with valid demographic data from the central person registry, and the data from 2 surveys, 15 years apart, that used the same survey instrument to assess suicidal thoughts. The major weakness is the small absolute number of completed suicides that results in low statistical power and makes it impossible to stratify analyses on several levels simultaneously, for example, sex and region.

## Conclusion

Suicide rates and the prevalence of suicidal thoughts remain high in Greenland but different regional trends point towards an increased marginalization between towns on the central West Coast, villages and East and North Greenland. Different temporal patterns call for different regional strategies for prevention.
